# Immune-and Metabolism-Associated Molecular Classiﬁcation of Ovarian Cancer

**DOI:** 10.3389/fonc.2022.877369

**Published:** 2022-05-12

**Authors:** Zhenyue Chen, Weiyi Jiang, Zhen Li, Yun Zong, Gaopi Deng

**Affiliations:** ^1^ The First Clinical Medical College, Guangzhou University of Chinese Medicine, Guangzhou, China; ^2^ College of First Clinical Medicine, Shandong University of Traditional Chinese Medicine, Jinan, China; ^3^ The Second Clinical Medical College, Guangzhou University of Chinese Medicine, Guangzhou, China; ^4^ Department Obstetrics and Gynecology, First Affiliated Hospital of Guangzhou University of Chinese Medicine, Guangzhou, China

**Keywords:** ovarian cancer, metabolic genes, subtypes, molecular characteristics, tumor

## Abstract

Ovarian cancer (OV) is a complex gynecological disease, and its molecular characteristics are not clear. In this study, the molecular characteristics of OV subtypes based on metabolic genes were explored through the comprehensive analysis of genomic data. A set of transcriptome data of 2752 known metabolic genes was used as a seed for performing non negative matrix factorization (NMF) clustering. Three subtypes of OV (C1, C2 and C3) were found in analysis. The proportion of various immune cells in C1 was higher than that in C2 and C3 subtypes. The expression level of immune checkpoint genes TNFRSF9 in C1 was higher than that of other subtypes. The activation scores of cell cycle, RTK-RAS, Wnt and angiogenesis pathway and ESTIMATE immune scores in C1 group were higher than those in C2 and C3 groups. In the validation set, grade was significantly correlated with OV subtype C1. Functional analysis showed that the extracellular matrix related items in C1 subtype were significantly different from other subtypes. Drug sensitivity analysis showed that C2 subtype was more sensitive to immunotherapy. Survival analysis of differential genes showed that the expression of PXDN and CXCL11 was significantly correlated with survival. The results of tissue microarray immunohistochemistry showed that the expression of PXDN was significantly correlated with tumor size and pathological grade. Based on the genomics of metabolic genes, a new OV typing method was developed, which improved our understanding of the molecular characteristics of human OV.

## 1 Introduction

Ovarian cancer (OV) is a heterogenous gynecological disease with high mortality rate in the women (1–3). The treatment of OV is based on surgical debulking or chemotherapy with carboplatin and paclitaxel (4, 5). The unelucidated genetic heterogeneity of OV complicates its diagnosis and the development of appropriate therapeutics approaches. Thus, it is vital to stratify OV patients based on molecular characteristics.

In recent years, the accumulation of genomic data has incited researchers to classify diseases according to the distinct profiles of their molecular characteristics and their correlation with clinical features (6–10). In the past, different classification systems were proposed for OV. The basic classification is the classification based on morphological characteristics, but this classification approach does not provide substantial information for the prognosis and treatment of OV patients. For this reason, researchers have endeavored to identify the different molecular subtypes of OV (11). Studies based on RNA-seq data have allowed the classification of patients with serous OV into nine molecular subtypes differing in immunologic activity, MAPK signaling pathway, mesenchymal development, and hormonal metabolism (12). Other investigators have classified high-grade serous OV into 4 subtypes including C1 (mesenchymal), C2 (immunoreactive), C4 (differentiated) and C5 (proliferative) (13–15). Other classifications of high-grade OV based on hormone receptor expression have provided a clinically applicable molecular subtype classification (16). OV classification based on gene expression profiles, gene methylation, and metabolic profile have also been developed (17–19). Nevertheless, all these efforts have not yet been able to definitively overcome the challenge imposed by the pathological severity of OV.

Metabolic genes have been reported as key players in human cancers. Especially, studies have shown that metabolic genes play a major role in the tumorigenesis and progression of cancers as it was reviewed previously (20). Different studies have indicated that the classification of diseases based on metabolic genes can help understand the genetic diversity of human diseases such as hepatocellular carcinoma (21) and colorectal cancer (22) and their clinical implications. However, only one study has proposed the classification of OV patients based on 594 energy-metabolism related genes (23). A classification based on a large set of metabolic genes and an integrative characterization of the identified subtypes is needed, which may be advantageous for diagnosis and therapeutic purposes.

For this reason, our present study attempted to establish a classification based on the expression of a large set of metabolic genes. Our results showed that these genes have a considerable influence on the status of patients with OV. We were able to classify and highlight the immunological, mutational and transcriptomic profiles of patients with a favorable prognosis and those with a severe prognosis. In addition, the sensitivity of each of the identified subtypes to anticancer therapy was established. Therefore, we believe that the results of our study may lead to new approaches in the effective management of OV patients.

## 2 Materials and Methods

### 2.1 Data Acquisition

We downloaded the processed mRNA expression data of ovarian serous cystadenocarcinoma (OV) cohort from the TCGA database (https://portal.gdc.cancer.gov/). A total of 377 specimens with complete survival information were collected. The mutation data was obtained by downloading the SNP data of OV cohort while the GISTIC2 copy number data of this cohort was acquired from GDAC FireBrowse (http://firebrowse.org/). Predicted neoantigens for the OV cohort were retrieved from a previous analysis of the TCGA dataset (24). The GSE63885 (n=75 samples) and GSE17260 (n=110 samples) datasets were downloaded from the GEO database for gene expression profile and survival analyses of the corresponding patients in external validation. The combat function in the R SVA library was used for batch effect correction of merged datasets.

### 2.2 Classification of OV Subtypes

The flowchart of the study design is as indicated in **Additional Figure S1**. The previously published metabolism-related genes (25) were retrieved and used for subsequent NMF clustering. Then, the R library “survival” was used for performing Cox regression analysis to evaluate the correlation between all candidate genes and overall survival (OS) of OV patients. Subsequently, the NMF package was used to perform an unsupervised NMF clustering method. The same candidate genes were used to apply the NMF clustering method to the two GEO external validation datasets. The k value at which the correlation coefficient begins to decrease was selected as the optimal number of clusters. SubMap analysis (Gene Pattern) is a method for evaluating the similarity of classification between patient cohorts based on expression profiles; this method was used to determine whether the subcategories identified in the above datasets were relevant. Then, based on the t-SNE method, the mRNA expression data of the above-mentioned metabolic genes were used to verify the subtype assignment.

### 2.3 Immune Cell Infiltration

The MCP algorithm was used to evaluate eight immune (immune cell populations: T cells, CD8+T cells, natural killer cells, cytotoxic lymphocytes, B cell lines, monocyte cell lines, and marrow cells, dendritic cells and neutrophils) and two non-immune stromal cell populations (endothelial cells and fibroblasts). In addition, the ssGSEA analysis, which calculates an enrichment score indicating the degree to which genes in a specific gene set are coordinated in a single sample, was also used to estimate immune infiltration. The GSVA-R software package was used to estimate six other immune cell populations, including regulatory T cells (Treg), helper T cells 1 (Th1), helper T cells 2 (Th2), and helper T cells 17 (Th17), central memory T cells and effective memory T cells (Tem), helper T cell (TFH), gamma delta T cells (Tgd). In addition, the ESTIMATE algorithm was used to calculate the immune score and matrix score, which can reflect the richness of the genetic characteristics of the matrix and immune cells.

### 2.4 Generation and Performance Verification of Ovarian Cancer Classifier

The LIMMA library in R was used to analyze differentially expressed genes (DEGs) among OV subtypes; the selection was based on the condition of corrected P<0.05 and log_2_FC greater or equal to 2. Only genes whose expression were significantly different in all three possible comparisons were considered subtype-specific genes. The top 30 genes with the largest log_2_FC value in each subcategory were further selected to build a prediction model, and a 90-gene classifier was generated. Then, NTP algorithm was used to predict the sample subtypes based on the expression of the 90 genes in validation GEO datasets, followed by comparison of the classification results based on the NMF algorithm.

### 2.5 Prediction of Immunotherapy and Targeted Therapy Responses of Subtypes

By measuring the similarity of gene expression profiles between the subtypes obtained in the present study and previously published SubMap analysis (gene pattern) of gene profiles in patients with melanoma immunotherapy, we indirectly predicted the efficacy of immunotherapy on the predicted subtypes. In addition, based on the largest pharmacogenomics database (GDSC Cancer Drug Sensitivity Genomics Database, https://www.cancerrxgene.org/), we used the R library “pRRophetic” to predict the chemotherapy sensitivity of each tumor sample. The regression method was used to obtain the IC50 estimated value of each specific chemotherapy drug treatment, and the GDSC training set was used to perform 10 cross-validation tests to test the regression and prediction accuracy.

### 2.6 Tissue Microarray and Immunohistochemistry

Tissue chips were purchased from Shanghai Outdo Biotech Co.,Ltd. The chip number is HOvaC070PT01 include 69 cases. Immunohistochemical assay used the CXCL11(Affinity, Cat#DF9917) and PXDN (Cloud-Clone Corp, Cat#PAM070Hu01).The dilution of CXCL11 antibody is 1:200 and that of PXDN antibody is 1:100. The total score is the product of “staining intensity score” and “staining positive rate score” which represents CXCL11 and PXDN expression. According to the total score > 5 or < 5, they were divided into high expression group or low expression group. Use of all human samples was approved by the committee for ethical review of research involving human subjects at Shantou University and Jinan University.

### 2.7 Analytical Method of Immunohistochemistry

Use the tissue slice digital scanner or imaging system to collect the scanned documents or images on the immunohistochemical section, automatically read the tissue measurement area by using the Seville image analysis system, and analyze and calculate the number of weak, medium and strong positive cells in the measurement area (negative without coloring, 0 point; weak positive light yellow, 1 point; medium positive brownish yellow, 2 points; strong positive brownish brown, 3 points), the total number of cells and the positive cumulative optical density IOD value, Positive pixel area, tissue area mm². The following results were calculated to reflect the degree of positivity. The following indexes can be selected according to the section to evaluate the intensity of positive cells.

#### 2.7.1 Positive Rate

Number of positive cells/total number of cells. Reflect the number of positive cells.

#### 2.7.2 Positive Cell Density

Number of positive cells/area of tissue to be tested. It reflects the number of positive cells per unit area and is mostly used to evaluate the distribution and quantity of certain types of cells, such as the distribution and quantity of lymphocytes (CD3, CD4, CD8, etc.) in tumor tissue.

#### 2.7.3 Average Optical Density

Positive cumulative optical density IOD value/positive area. Reflect the average depth of positive signals.

#### 2.7.4 H-Score

The abbreviation of histochemistry score. It is a histological scoring method to deal with immunohistochemistry. It converts the positive number and staining intensity in each section into corresponding values to achieve the purpose of semi quantitative staining of tissues. H-Score(H-SCORE=∑(pi × i)=(percentage of weak intensity cells × 1)+(percentage of moderate intensity cells × 2)+(percentage of strong intensity cells × 3) In the formula, I represents the grade of positive cells: negative without coloring, 0 point; Weak positive light yellow, 1 point; Medium positive brownish yellow, 2 points; 3 points for strong positive tan. PI is the percentage of positive cells). H-score is a value between 0 and 300. The larger the value, the stronger the comprehensive positive intensity.

#### 2.7.5 Positive Score

The average positive intensity of the measurement area is 0,1,2,3 points: negative without coloring, 0 point; Weak positive light yellow, 1 point; Medium positive brownish yellow, 2 points; 3 points for strong positive tan. Score the positive rate of cells: 0-5% is 0, 6% ~ 25% is 1, 26% ~ 50% is 2, 51% ~ 75% is 3, and > 75% is 4. The positive comprehensive score is the staining intensity value × Positive cell ratio score. The larger the data, the stronger the comprehensive positive intensity.

### 2.8 Statistical Analysis

All statistical analyses were performed using the R language software (version 4.0). Survival analysis was performed by Kaplan-Meier method, and the log-rank test was used for comparison. Univariate Cox proportional hazards regression model was used to estimate the hazard ratio of univariate analysis. All statistical tests were two-sided, and p<0.05 was retained as statistical significance treshold.

## 3 Results

### 3.1 Identification of Novel OV Subtypes Based on Metablism-Related Genes

**Figure 1:**
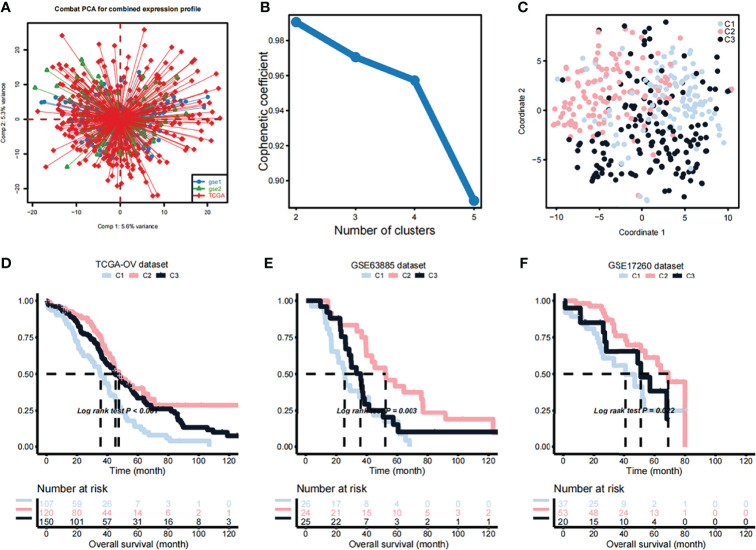
Identification of OV subtypes based on metabolic genes. **(A)** Combat principal component analysis (PCA) was performed to identify the distribution of the expression profiles of genes in the dataset merged TCGA and 2752 metabolism-related genes. **(B)** Cophenetic plot indicating the cophenetic coefficient in function of the number of cluster components. **(C)** Two-dimensional t-SNE distribution pattern distribution in the identified subtypes. **(D)** Differences in the survival probabilities of patients in the identified subtypes based on the TCGA dataset. **(E)** Differences in the survival probabilities of patients in the identified subtypes based on the GSE63885 dataset. **(F)** Differences in the survival probabilities of patients in the identified subtypes based on the GSE17260 dataset.

A list of 2752 previously published metabolism-related genes (these genes encode all known human metabolic enzymes and transport proteins) were used as input in the NMF analysis. After merging of TCGA and 2752 metabolism-related genes and correction of the batch effects using the Combat PCA algorithm (**Figure 1A**), Cox regression analysis was applied and a total of 177 prognostic-related candidate genes were identified (**Additional File S1**). Next, the NMF consensus clustering method was used for OV classification based on the expression profiles of the above 177 candidate genes. The clustering was performed on the TCGA dataset, and after a comprehensive consideration, k=3 was selected as the optimal number of clusters based on the cophenitic coefficient (**Figure 1B**). For k=3, we performed dimensionality reduction analysis by t-SNE and found that the distribution of the subtypes was largely consistent with the two-dimensional t-SNE distribution pattern **(** **Figure 1C**). We observed significant prognostic differences among the three subtypes in the TCGA data set (**Figure 1D**). Compared with C2 and C3, C1 had a shorter median survival time (**Figure 1D**). Subsequently, the OV samples from the GEO datasets were used in independent verification, using the aforementioned k=3 classification, which also revealed three different molecular subtypes. Obvious differences between the three subtypes were observed in the GSE63885 (**Figure 1E**) and GSE17260 (**Figure 1F**) datasets, and the overall survival (OS) of the C1 subtype was significantly shorter than that of C2 and C3 subtypes.

### Metabolic and Immune Characteristics Among OV Subtypes

**Figure 2:**
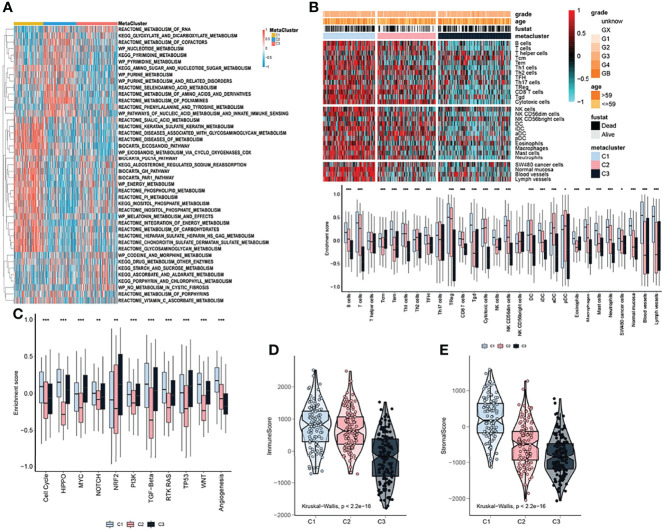
Metabolic and immune characteristics of OV subtypes. **(A)** Heatmap showing that the ssGSEA algorithm quantitatively identified differential metabolic pathways among the identified subtypes. **(B)** Heatmap (upper pane) and boxplot (lower pane) indicating the differences in immune characteristics of the identified subtypes. **(C)** Boxplot showing the differences in pathway enrichment scores among the three identified subtypes. **(D)** Immune score differences among the identified subtypes. **(E)** Stromal score differences among the identified subtypes. The statistical difference was compared through the Kruskal–Wallis test, and the P values are labeled above each boxplot with asterisks ( *P < 0.05, **P < 0.01, ***P < 0.001).

Considering that the subtyping of OV samples was based on metabolism-related genes, we used the ssGSEA algorithm to quantitatively analyze the metabolic process. We uncovered multiple metabolic pathways that were significantly different among the three subtypes. Such as, the pathways predominant in the C1 subtype were the inositol phosphate metabolism (some specific metabolic genes: ALDH6A1, CDIPT, IMPA1, IMPA2), reactome inositol phosphate metabolism (some specific metabolic genes: CALM1, IMPA1, IMPA2, INPP1), wp melatonin metabolism and their effects (some specific metabolic genes: AANAT, ACHE, ADRB1, APOE) were significantly higher than the other two subtypes. Also, there were some pathways, certain differences between the C2 and C3 subtypes, such as kegg glyoxylate and dicarboxylate metabolism (some specific metabolic genes: ACO1, ACO2, AFMID, CS), kegg pyrimidine metabolism (some specific metabolic genes: AK3, CAD, CANT1, CDA) and wp pyrimidine metabolism (some specific metabolic genes: CAD, CDA, CMPK1, CMPK2) (**Figure 2A**). We further explored the immune characteristics between different subtypes. First, the immune process was quantified using the ssGSEA algorithm and differential analysis was performed to discover subtype-specific immune characteristics. The results showed that the immune characteristics of the three subtypes were clearly classifiable, and differences in the enrichment scores of B cells, Th1 cells, Th2 cells, TFH, Tgd, NK CD56dim cells, macrophages, mast cells, normal mucosa, blood vessels, lymph vessels were significant between C1 and other subtypes (**Figure 2B**). In addition, C1 had higher cell cycle, RTK RAS, WNT, and angiogenesis pathway activation scores than C2 and C3 (**Figure 2C**). Moreover, the ESTIMATE algorithm was used to calculate the immune scores and significant differences in the immune scores were observed between the three groups with the immune score of C3 being significantly lower than that of C1 and C2 (**Figure 2D**). The immune score of C1 was the highest (**Figure 2D**). Meanwhile, the stromal scores showed a tendency similar to that of the immune score (**Figure 2E**).

### 3.3 Immune Checkpoints in OV Subtypes

**Figure 3:**
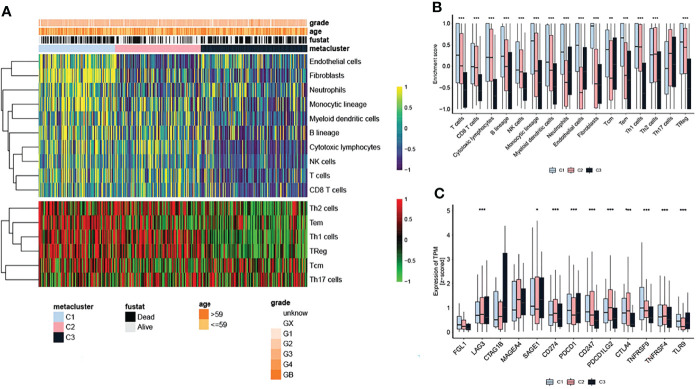
Immune checkpoints in OV subtypes. **(A)** Heatmap showing the level of immune infiltration in the different subtypes. **(B)** Differences in the enrichment score of infiltrated immune cells among the three subtypes. **(C)** Expression of immune checkpoint genes among the three subtypes. The statistical difference was compared through the Kruskal–Wallis test, and the P values are labeled above each boxplot with asterisks ( *P < 0.05, **P < 0.01, ***P < 0.001).

Due to the significant differences in the immune scores between the subtypes, the MCP and ssGSEA algorithms were further used to conduct in-depth research on immune infiltration. The abundance of 16 immune-related cell types was calculated using MCP counter and ssGSEA algorithm and displayed in the heatmap (**Figure 3A**). The results showed that there were significant differences in the level of immune infiltration between the three subtypes (**Figure 3A**), suggesting that the immune characteristics of the three subtypes are reproducible. In addition, the enrichment score of T cells, B lineage, monocytic lineage, myeloid dendritic cells, neutrophils, endothelial cells, fibroblasts, Tcm, Tem and TReg in C1 were higher than in C2 and C3 subtypes.(**Figure 3B**). We further studied the association between the subtypes and the expression of 12 potentially targetable immune checkpoint genes, which were selected based on current inhibitor drugs in clinical trials or drugs approved for specific cancer types, and the results indicated remarkable differences in the expression of these genes between the three subtypes, except for FGL1, CTAG1B and MAGEA4 (**Figure 3C**). The immune checkpoint genes TNFRSF9 showed higher expression in C1 compared to the other two subtypes (**Figure 3C**).

### 3.4 Correlation Between Clinical Symptoms and Signaling Pathways With OV Subtypes

**Figure 4:**
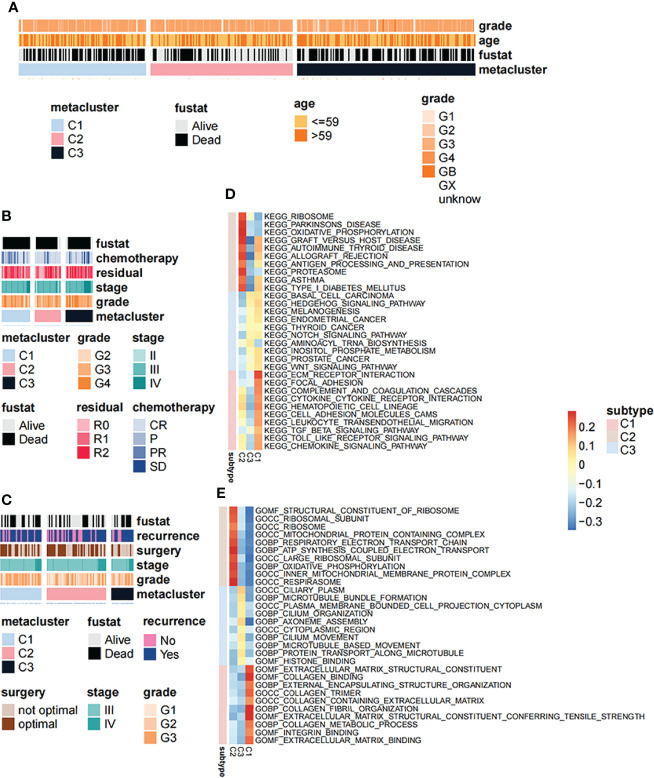
Correlation between clinical symptoms and signaling pathways with OV subtypes. **(A)** Correlation of OV subtypes with age, pathological stage, grade and survival of OV patients in TCGA dataset. **(B)** Correlation of OV subtypes with age, pathological stage, grade and survival of OV patients in GSE63885 dataset. **(C)** Correlation of OV subtypes with age, pathological stage, grade, tumor recurrence and survival of OV patient in GSE17260 dataset. **(D)** Differences in gene ontology enrichment functional terms among the three subtypes. **(E)** Differences in KEGG enrichment functional pathways among the three subtypes.

We further explored the relationship between different subtypes and clinical symptoms in order to reveal their clinicopathological characteristics. Based on the TCGA dataset, the results of the chi-square test showed that the correlation between clinicopathol ogical characteristics and OV subtypes in the TCGA cohort was not obvious (**Figure 4A**). However, in validation set 1 (**Figure 4B**), the proportion of chemotherapy and residuals in the three groups was consistent with the results of KM analysis in **Figures 1E–G** whereas in validation set 2, the grade was higher in C1 (**Figure 4C**). In addition, we used GSEA to analyze the differences in signaling pathways between the subtypes. GO analysis results showed that the C1 subtype was mainly enriched in the extracellular matrix structural constituent and collagen binding signaling pathways while the C2 subtype was mainly enriched in the structural constituent of ribosome and ribosomal subunit signaling pathways (**Figure 4D**). The C3 subtype was mainly enriched in microtubule bundle formation, plasma membrane and cell projection cytoplasm signaling pathway (**Figure 4D**). KEGG analysis results showed that the C1 subtype was mainly enriched in the ECM-receptor interaction and focal adhesion signaling pathways (**Figure 4E**). The C2 subtype was mainly enriched in the ribosome and oxidative phosphorylation signaling pathways while the C3 subtype was mainly enriched in basal cell carcinoma and hedgehog signaling pathway signal pathway (**Figure 4E**).

### 3.5 Mutation Landscape of OV Subtypes

**Figure 5:**
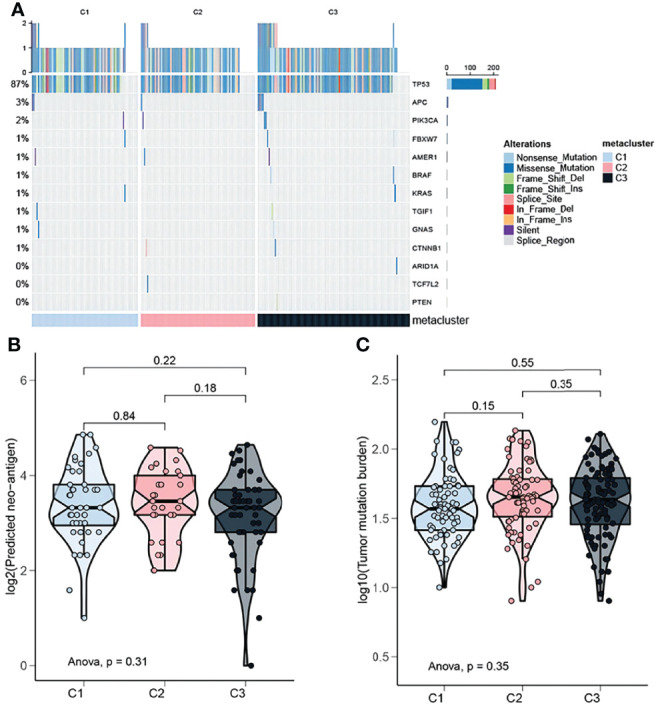
Mutation landscape of OV subtypes. **(A)** Oncoplot showing the distribution of mutated genes in the identified OV subtypes. **(B)** Proportion of neoantigens in the different subtypes. **(C)** Proportion of tumor mutation burden in the different subtypes.

The instability of the genome promotes the accumulation of mutations in cancer cells and leads to the rapid evolution of the cancer genome in response to the tumor microenvironment and treatment-induced related stresses during evolution (26). Detecting and characterizing these tumor somatic mutations has become an important means to analyze the occurrence and development of tumors. In order to study the differences in the frequency of somatic mutations among OV subtypes, and to observe different mutation patterns among OV subtypes, the somatic mutation data from the TCGA database was analyzed. The mutation characteristics among the three subtypes were shown in **Figure 5A**. Most of the mutations in the three subtypes were nonsense mutation, but C2 has fewer mutated genes than C1 and C3 (**Figure 5A**). On the other hand, in a multi-group comparison, the results showed that the C2 subtype had a higher number of neoantigens than the C1 and C3 subtypes but not significant (**Figure 5B**, P>0.05). Moreover, the tumor mutation burden in the C2 subtype was slightly higher than that of the C1 and C3 subtypes but not significant (**Figure 5C**, P>0.05).

### 3.6 Performance Verification of Immune Classifiers

**Figure 6:**
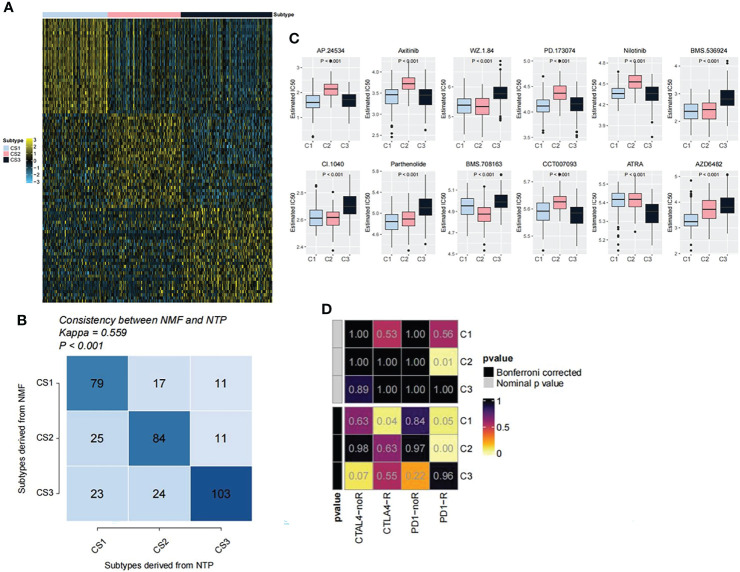
Performance verification of immune classifiers. **(A)** Heatmap showing the classification of the 90-gene classifier for OV subtypes. **(B)** Correlation between NTP classification and NMF classification. **(C)** Drug sensitivity analysis of OV subtypes. **(D)** Prediction of the sensitivity of the three subtypes to anti-tumor immunotherapy.

After comprehensively considering accuracy and clinical application potential, the top 30 genes with the largest log_2_FC values in each subtype were selected for the development of subtype classification. Therefore, a 90-gene classifier was generated and visualized (**Figure 6A**). Subsequently, the 90-gene classifier was used to repeat the subtype prediction in the GEO dataset. The results showed a consitency between the NMF and the NTP predicted clusters, confirming that the subtypes were predictable based on the 90-gene classifier (**Figure 6B**). In addition, based on the pRRophetic oftware package, we predicted the sensitivity of each OV subtypes to anticancer drugs. The results showed that the C2 subtype had a lower IC50 for BMS.708163 than C1 and C3, while the C1 subtype had a lower IC50 for AZD6482 than other two subtypes (**Figure 6C**). Moreover, we further based on the data set of melanoma immunotherapy to predict the sensitivity of the three subtypes to anti-tumor immunotherapy. The results showed that the C2 subtype was more sensitive to immunotherapy (**Figure 6D**).

### 3.7 Significant Difference Gene Analysis

**Figure 7:**
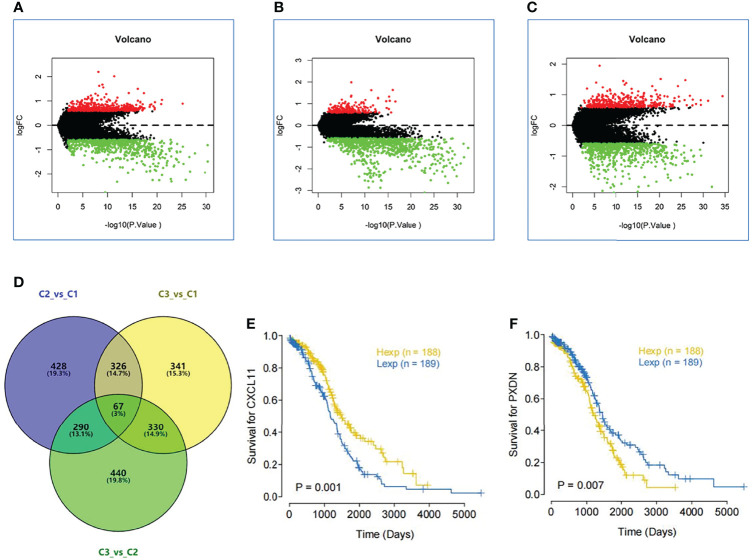
Significant difference gene analysis. **(A)** Different expression analysis among subgroups C1 vs C2. **(B)** Different expression analysis among subgroups C3 vs C1. **(C)** Different expression analysis among subgroups C3 vs C2.(|log_2_FC| > 0.585 and. p value < 0.01). **(D)** Wayne analysis shows mount of different expression gene in three subgroups. **(E)** The survival curve of the different expression of CXCL11. **(F)** The survival curve of the different expression of PXDN.

Through the previous subtype analysis, we divided the samples into three subgroups: C1, C2 and C3. We performed difference analysis among subgroups (C1_vs_C2(**Figure 7A**), C3_vs_C1(**Figure 7B**) and C3_vs_C2(**Figure 7C**) respectively) (|log_2_FC| > 0.585 and. p value < 0.01). According to the Wayne analysis (**Figure 7D**), 1111 genes were differentially expressed between C2 and C1 groups; 1064 genes were differentially expressed between C3 and C1 groups; 1127 genes were differentially expressed between C3 and C2 groups. In the three differential analysis, 67 genes were differentially expressed at the same time. Finally, we analyzed the survival of these 67 genes and found that the expression of two genes was very significant in the survival analysis (p value < 0.01). The sequence is CXCL11(**Figure 7E**) and PXDN (**Figure 7F**).

### 3.8 Association Between the Expression of CXCL11 and PXDN and the Progression of OV

**Figure 8:**
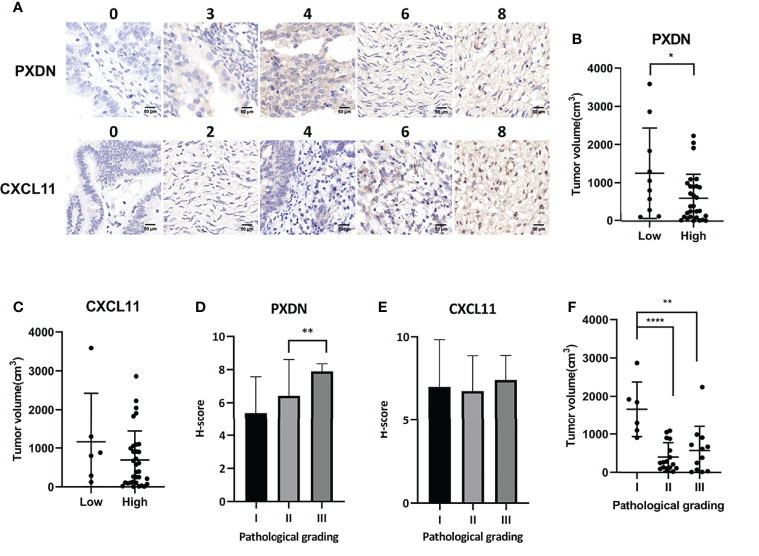
Association between the expression of CXCL11 and PXDN and the progression of OV. **(A)** We divided two subgroups in each gene: PXDN high group(n=51); PXDN low group(n=14); CXCL11 high group(n=54); CXCL11 low group(n=11). Results shows the staining corresponding to different scores. **(B)** The relationship between tumor size and the expression of PXDN. **(C)** The relationship between tumor size and the expression of CXCL11. **(D)** The correlation between H-score and Pathological grade of PXDN. **(E)** The correlation between H-score and Pathological grade of CXCL11. **(F)** There was an inverse correlation between tumor size and pathological grade. Error bars, SD. *P < 0.05, **P < 0.01, ****P < 0.0001.

We used immunohistochemistry to stain the tissue chip, and then scored according to the staining intensity and staining area. **Figure 8A** shows the staining corresponding to different scores. After comparing the score with the patient information, we found that the expression of PXDN was inversely proportional to the tumor size (**Figure 8B**) and H-score increases with the increase of pathological grade positively correlated with the pathological grade (**Figure 8D**). The expression of CXCL11 was inversely proportional to the tumor size (**Figure 8C**), while the H-score was no correlation with the pathological grade (**Figure 8E**). Furthermore, it was found that there was an inverse correlation between tumor size and pathological grade (**Figure 8F**).

## 4 Discussion

The present study was devoted to the discovery of novel OV subtypes and their clinical value based on metabolic features. A list of 2752 metabolism-related genes, previously reported in the literature, were used as a seed for NMF clustering. We identified three OV subtypes based on our clustering approach and on the expression of 177 prognostic-related metabolic genes identified by cox regression analysis. C1 had a shorter median survival time, higher cell cycle, RTK, RAS, WNT, and angiogenesis pathway activation scores and the pathway predominantly affected in this subtype was the inositol phosphate metabolism pathway, and the inositol phosphate metabolism pathway involved in cell proliferation, migration and phosphatidylinositol-3-kinase (PI3K)/Akt signaling, play a crucial in cancer and are frequently dysregulated in cancer (27). According to KEGG pathway map analysis, we found that the inositol phosphate metabolism pathway which C1 enriched in, aldehyde dehydrogenase 6 family member A1 (ALDH6A1) overexpressed the Acetyl coenzyme A (CoA). Researcher found that in highly proliferating cancer cells, the intermediate of the TCA cycle is rapidly consumed (28). In the inositol phosphate metabolism pathway, the CoA participates in the next pathway TCA cycle which increases the synthesis of the intermediate of the TCA cycle and accelerates cell proliferation. While the Glyoxylate and dicarboxylate metabolism pathway which C2 enriched in, acyl-CoA synthetase short chain family member 2(ACSS2) was involved in the activation of Acetyl coenzyme A and the fatty acid degradation pathway can indirectly promote the process and researchers have found that ACSS2 activates the CoA under metabolic stress to encourage the tumor cells to use acetic acid as an additional nutrient source that allows the tumor cells to adapt to the harsh metabolic environment and keep the tumor cells alive (29). These processes maybe work together to promote OV occurrence. Significant differences in the enrichment scores of B cells, Th1 cells, Th2 cells, TFH, Tgd, NK CD56dim cells, macrophages, mast cells, normal mucosa, blood vessels and lymph vessels were observed between C1 and the other subtypes which was reflected by higher immune and stromal scores associated with C1. Differences in the expression of immune checkpoint genes were recorded between the three subtypes, with TNFRSF9 being significantly and highly expressed in C1 compared to the other subtypes. The pathways mostly in play were ECM-receptor interaction and focal adhesion signaling pathways in the C1 subtype, ribosome and oxidative phosphorylation signaling pathways in the C2 subtype and basal cell carcinoma and hedgehog signaling pathway signal pathway in C3 subtype. Differences in gene mutations were observed among the three subtypes. Moreover, the C1 subtype was more sensitive to drugs AZD6482, and C2 subtype was more sensitive to drugs BMS.708163, this evidence may provide a hint for future treatment.

Immune score has been reported as an important prognostic and indicator of chemosensitivity for OV (30, 31). Here, the three subtypes identified showed significant differences in immune score, and the highest immune score was recorded in C1. We anticipated that C1 was the highest risk score subtype, which was in corroboration with previous findings indicating that higher risk score subtypes are correlated with higher immune score (32–34). This observation was further supported by the increase of immune cells in C1 subtype compared to C2 and C3. Indeed, the increased proportions of B cells, CD8+ T cells, NK cells, cytotoxic lymphocytes, Neutrophils, monocytic lineage, Th2 cells, Tem and Treg cells in the C1 subtype indicated increased recruitment of immune cells to counteract the pathological status in OV patients. Moreover, obviously higher levels of fibroblasts and endothelial cells were observed in the C1 subtype of OV. Studies have conveyed that the stromal compartment is also associated with the prognostic outcome (35–37), which supports the increased stromal score observed in the present study. As important elements of the stromal compartment, the cancer-associated fibroblasts (CAFs) constitute a determinant repertoire in the regulation of the cancer cell proliferation, invasion and metastasis and are associated with poor prognostication of tumors, especially after treatment (37–42). In addition, endothelial cells are known for their involvement in cancer angiogenesis. The increased levels of endothelial cells in the C1 subtype further confirmed the high-risk score status of this subtype. The other C2 and C3 groups could be classified in the medium and low risk score subtypes, but the heterogeneity of cell enrichment scores in these subtypes renders this conclusion somehow difficult.

TNFRSF9 is an important check point in cancer immunotherapy. However, as stated in previous studies, the significance of TNFRSF9 in cancer is not clear, but it was suggested to be immunosuppressive (43–46). Increased expression of TNFRSF9 was observed in platinum resistant ovarian tumors (27, 47, 48), which implies that this gene may promote tumor progression. In this study, we found that the expression level of TNFRSF9 was the highest in the C1 subtype, which make us suspected the high expression of TNFRSF9 may have something to do with the low survival of C1 subtype.

The mechanism OV in C1 subtype patients chiefly involved inositol phosphate metabolism pathway, indicating poor prognosis when contrasted with C2 and C3. Indeed, genes participating in the inositol phosphate metabolism pathway have been incriminated in cancer risk and known to be regulators of cell cycle, metastasis and PI3K/Akt signaling (49).

The enrichment in metabolic signatures showed significant enrichment of a large number of pathways in C1 patients, indicating that this subtype may be more sensitive to metabolic therapies, which have been proven efficient in cancer chemotherapy (50). However, due to the huge number of enriched pathways, additional experimental works are needed to confirm the most important pathways. Up to now, several metabolism-targeting drugs for OV have been developed and evaluated in clinical trials. These drugs include those targeting the hexokinases in glycolysis pathway (2-deoxy glucose, 3-bromopyruvate, lonidamine, methyl jasmonate), PDK1 in Krebs cycle (dichloroacetate), LDH and Bcl-xL in the Bcl-xL pathway (Gossypol) and mitochondria complex I in the mitochondrial respiration pathway (metformin) (51). However, the efficacy of these drugs is questionable due to their side effects or broad range of targets. Here, the classification of OV in different subtypes may potentiate metabolic therapies due to the accurate knowledge of metabolic profiles of OV patients. Especially, we found that drugs targeting genes in pathways such as energy metabolism, inositol phosphate metabolism, metabolism of carbohydrates, melatonin metabolism, glycosaminoglycan metabolism, keratan sulfate/keratin metabolism, eicosanoid metabolism *via* cyclo-oxygenases cox, PGC1a pathway, aldosterone regulated sodium reabsorption, phospholipid metabolism and PAR1 pathway may be efficient for the treatment of patients classified in the C1 OV subtype identified in this study. For the C2 OV subtype, drugs targeting the selenoamino acid metabolism, metabolism of amino acids and derivatives, metabolism of polyamines, phenylalanine and tyrosine metabolism, pathways of nucleic acid metabolism and innate immune sensing and metabolism of cofactors may be potential metabolic drugs. In tissue microarray staining, we found that the relationship between PXDN and tumor size and pathological grade showed an opposite trend, but the literature described that PXDN was positively correlated with tumor proliferation (52). This contradictory phenomenon may be due to the limited sample size of this study and need for more in-depth research on the influence of PXDN on tumor size and pathological grade in the future. CXCL11 shows no difference in the chip, possibly because the sample size is too small and needs to be increased to continue the analysis.

In pathway enrichment analysis, we found that ECM-receptor interaction pathway was mainly enriched in patients classified in the C1 subtype. This pathway is an integral part of malignant microenvironments and regulates homeostasis. Studies have implicated the dysregulation of ECM components in OV tumorigenesis and progression and suggested that the elucidation of ECM function in OV is important for developing effective biomarkers and novel drugs (53–57). Studies have also shown that the potential biomarkers of OV metastasis are enriched in ECM-receptor interaction pathway (57) among other pathways including PI3K/Akt signaling pathway which was also found implicated in the inositol phosphate metabolism as described above. ECM-receptor interaction pathway was also incriminated in the survival and prognosis of high-grade serous OV patients (58–60). ECM-Receptor pathway was similarly reported to be enriched in C1 in a previous classification of OV patients based on energy-metabolism related genes (51).

In conclusion, this proposed a metabolism-related molecular classification of OV patients and identified three subtypes characterized by active, medium, or low metabolic activities, respectively. C1 showed low survival probability while C2 showed high sensitivity to immune blockade and chemotherapy. C3 had high heterogeneity and may be further investigated. Our classification system may display predictive value for the prognosis of OV patients and discovery of novel therapies.

## Data Availability Statement

The original contributions presented in the study are included in the article/**Supplementary Material**. Further inquiries can be directed to the corresponding author.

## Ethics Statement

The studies involving human participants were reviewed and approved by Shanghai outdo biotech company. The patients/participants provided their written informed consent to participate in this study. Written informed consent was obtained from the individual(s) for the publication of any potentially identifiable images or data included in this article.

## Author Contributions

ZC and GD conceived and designed the experiments. WJ, ZL, and YZ analyzed the data and prepared the figures. ZC wrote the main manuscript text. All authors contributed to the article and approved the submitted version.

## Conflict of Interest

The authors declare that the research was conducted in the absence of any commercial or financial relationships that could be construed as a potential conflict of interest.

## Publisher’s Note

All claims expressed in this article are solely those of the authors and do not necessarily represent those of their affiliated organizations, or those of the publisher, the editors and the reviewers. Any product that may be evaluated in this article, or claim that may be made by its manufacturer, is not guaranteed or endorsed by the publisher.

## Supplementary Material

Supplementary Figure 1 The flowchart of the study design.

Additional File S1 List of potential prognostic genes.
