# Supplementary figures and images for: Immune-and Metabolism-Associated Molecular Classiﬁcation of Ovarian Cancer
Source: Front Oncol. 2022 May 12;12:877369. doi: 10.3389/fonc.2022.877369 (PMC9133421; doi:10.3389/fonc.2022.877369)

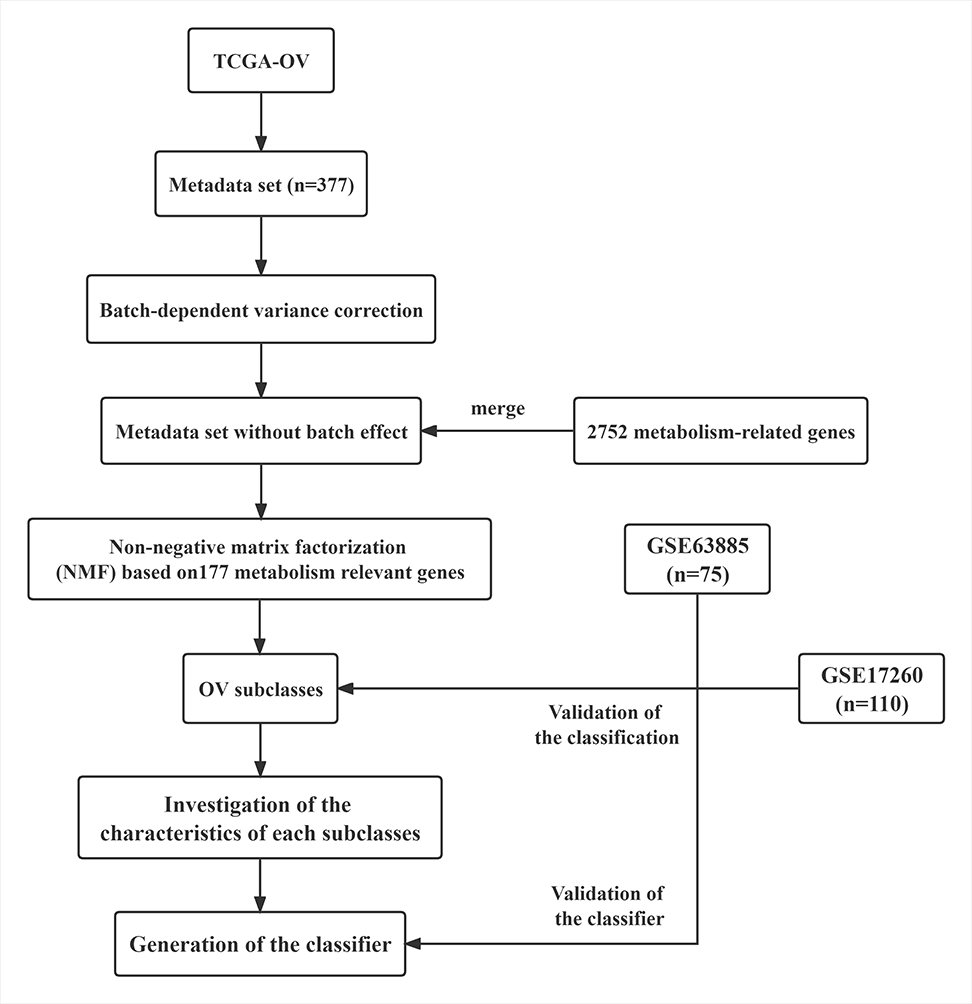

Supplement: Supplementary Figure 1 — The flowchart of the study design. [file Image_1.tif]
