# Supplementary material for: Immune-and Metabolism-Associated Molecular Classiﬁcation of Ovarian Cancer
Source: Front Oncol. 2022 May 12;12:877369. doi: 10.3389/fonc.2022.877369 (PMC9133421; doi:10.3389/fonc.2022.877369)
Supplement: Additional File S1 — List of potential prognostic genes. [file DataSheet_1.pdf]

	HR	pvalue	lower	upper
ABCA13	0.800523137704251	0.000827611631593836	0.702634862456502	0.912048815453605
ABCB10	0.880866691891203	0.0243592753704221	0.788772716643071	0.983713194576008
ASNSD1	0.878041797171544	0.0251891088907517	0.783535275711245	0.983947272674363
SCCPDH	0.886541358363556	0.0399209699686953	0.790325841034748	0.994470304880925
GNPDA2	0.85122818426426	0.00501345439581534	0.760653242793653	0.952588355535866
GNPTAB	1.14638105692826	0.015613818127963	1.0262069766457	1.28062813603117
PGM3	0.876798815154062	0.0201561476708518	0.784751813264291	0.979642415934956
GCH1	0.877297488543007	0.0173015492021306	0.787648007790621	0.977150803139545
NSDHL	0.886043143875001	0.0339778432289091	0.792289619979761	0.990890746275271
ACOT8	1.19377724058758	0.000887662389378504	1.07538739237224	1.32520067675445
NDUFA5	0.845532192143525	0.00432106570203584	0.753498482331329	0.948807070903519
NDUFC1	0.887468888729658	0.0329951810997012	0.795230613711295	0.990405820504527
NDUFV2	0.870644164988278	0.0182797107529495	0.776026846732598	0.97679772964006
NFS1	1.16478285321338	0.00509472415833026	1.04686483639397	1.29598306101602
AADAC	0.811233947059283	0.000373915778585689	0.722929590557571	0.910324498342658
EPHX2	0.882276029385592	0.0305914884707593	0.787589735874713	0.988345780260693
TPMT	0.871794795159696	0.0176792550219239	0.778367407796944	0.976436265514613
UGT2B28	0.874762788943304	0.0351030343718084	0.772396086634882	0.990696289326992
ACACB	1.13785663656437	0.0182653038832222	1.02214163591216	1.26667154519947
ACAD9	1.13514239737823	0.0244963775904283	1.01643671010642	1.26771125984882
ACSM3	0.852024135165153	0.00949047744011612	0.754918752245644	0.961620207134172
CYP4F22	1.16524587872813	0.0022474107352949	1.05636181936692	

1.28535311765301
ECH1 1.14984954845755 0.0134234297517515 1.02936007035335
1.28444265730472
LPL 1.12009090779176 0.0396170387113056 1.0054022862787 1.24786233216302
SCD5 1.13658009953119 0.0219614229007672 1.01867252851372
1.26813503504912
SHMT2 0.879883322690478 0.0250989492167117 0.786674814551405
0.984135563041147
ALDH18A1 0.878341850015565 0.0232360517099625 0.785256455030576
0.982461717501865
ALDH5A1 0.87653859386454 0.0271450288335164 0.779836745167075
0.985231731250899
GSTK1 0.885314213157578 0.0394948687935946 0.788386678191218
0.994158422129909
MGST2 0.880063444424046 0.0245730418020201 0.787296723813264
0.983760814423533
ALG13 0.887611263087976 0.0315184394358299 0.796218648414512
0.989494224895967
B3GALT1 1.11943765981415 0.0388777609754463 1.0057803503103
1.24593871198972
B4GALT5 1.19092100788766 0.00185038063194571 1.06686739776338
1.32939937053238
FUT8 0.855709525493829 0.0101381540936815 0.759867725514615
0.963639811817208
GALNT10 1.1989006651851 0.00153073441314246 1.07165294165442
1.34125774223348
GALNT6 0.835065691814091 0.00246031003923694 0.743113234854319
0.938396299430265
GLT8D1 0.885803039461858 0.0300413443538282 0.793892374504073
0.988354404096674
MGAT4C 1.10489409971544 0.0314031350180967 1.00893786036753
1.20997637172748
MGAT5 1.13150848995039 0.028009089070014 1.0134274141566
1.26334796646025
ST3GAL3 1.14531449864278 0.0181833069504163 1.02335606893373
1.28180732066027
ST3GAL6 0.864274845646136 0.0140368283425215 0.76931585782034
0.970954909122768
ST6GALNAC1 0.879518677783557 0.023975227886783 0.78675430757417
0.983220679090099
ST6GALNAC2 0.84023830424961 0.00194237029249914 0.752643520938155
0.938027616378396
DPM3 0.858685647159848 0.0122192528612433 0.762219708918169
0.967360240113506

PIGW	0.876962188810774	0.0284520852996676	0.77978375270223	0.986251224058857
GLB1L2	0.888558480834817	0.0371570842068325	0.795109487601535	0.992990507816893
HYAL2	0.860728878779421	0.0114534842089134	0.766265214198555	0.966837837653567
BHMT	1.13677103180055	0.0229856126862135	1.01784458790444	1.26959301458919
BHMT2	1.13666426340396	0.0282943005062494	1.01371481382305	1.27452576413191
ALDOC	1.1172862075637	0.0381168503114083	1.00609591606792	1.24076487109784
PFKL	1.13587071101185	0.0223709331442825	1.01823926987739	1.26709144923269
PGM2L1	0.849705093855494	0.00535304028506086	0.75768716210887	0.95289821793263
HAO1	1.12770779332963	0.0303510662256243	1.01147163800339	1.25730155879281
HS3ST3A1	1.11603194933736	0.0346783964438995	1.00793854234459	1.23571751611411
HS3ST5	1.16769989377419	0.00929235088798304	1.03895413237025	1.31239965214783
AMDHD1	1.14296430071111	0.0128704445225271	1.02873665889483	1.26987541603063
ALOX12	1.12476467982618	0.0328250619947404	1.00964578307035	1.25300932881364
HPGD	0.854915984577484	0.0131722592794688	0.75526953329862	0.967709285841287
PTGIS	1.13969116686625	0.0260540383848087	1.01571869968815	1.27879496186469
PTGR2	0.86205406725876	0.0129427849808154	0.766825181054497	0.969109039762424
INPP1	0.883843403361314	0.0343877479985722	0.788302929297588	0.990963159760646
ITPKC	1.15219235149839	0.00806642803018687	1.03754782643236	1.2795045982759
ATP2C2	0.859584641651253	0.0119868256587989	0.763886521476113	0.967271623977486
CACNA1C	1.19637313673331	0.000284710327782281	1.08595080086451	1.31802350636664
CACNA1G	1.17027425622089	0.00994816965008129	1.03839689140527	1.3189001682391
CACNG1	1.13517162978365	0.0136142760841041	1.02641187121094	1.25545569493986

CATSPER1	1.13357745618305	0.0279492255644964	1.01367058684744	1.26766808255019
CLCC1	0.871103885124254	0.0165104152584927	0.778171938972175	0.975134081140008
CLCN7	1.1377862873157	0.0174450572680952	1.02291455303918	1.26555794103953
GABRA3	1.11819429340935	0.0291206613786007	1.01142576002453	1.23623356971145
KCNK2	1.11738287806176	0.0393998507369242	1.00540361212383	1.24183410635271
KCNK5	0.864070807765179	0.0132146948498478	0.769771584951204	0.969921955328211
KCNS1	1.15672882974205	0.00608379193172777	1.04244781838911	1.28353818958924
KCTD15	0.882046085619339	0.0356649922372556	0.784575955874691	0.991625210192724
KCTD4	1.15037128256331	0.0139841674245772	1.02877496484165	1.28633970787775
SCN1A	1.13131231079108	0.0360001107313895	1.0080910967573	1.26959512752802
SCN1B	1.18328748024173	0.00305785134502628	1.0585806897477	1.32268543575129
SLC12A1	0.861481543840158	0.0269401133930987	0.754883651764348	0.983132233215854
SLC12A9	1.13058942709457	0.0286523277487375	1.01288625943917	1.26197037500122
SLC39A13	1.15805293399692	0.01886319008041	1.02455900432816	1.30894032678797
SLC39A5	0.874595228826469	0.0198643239530443	0.781327183222161	0.978996802762626
SLC41A3	1.12137183440959	0.0395837183220312	1.00547537748122	1.25062713535281
MAGT1	0.858787059012075	0.00603830178980039	0.77035489979053	0.95737070397962
ORAI3	0.868165396364334	0.0134506535847642	0.776094806273446	0.971158612777626
TRPC5	1.17272175010751	0.00108578425339934	1.06582721807664	1.29033700758459
TRPM2	1.13092391501133	0.028496136610777	1.01302710833162	1.26254163489361
TRPM7	1.16785518570885	0.00837061458056517	1.0406313303329	1.31063297349574
TRPV4	1.14882822225817	0.0136709697505196	1.02887039255207	1.28277214876711

ME1	0.865810581709273	0.016087834915971	0.769956244347192	0.973598134833392
ME2	0.882089899820117	0.0355250416495032	0.784721022876817	0.991540392931215
PC	1.12235790771693	0.0312341072596639	1.01045635044684	1.24665184444402
CHKA	1.14103171863767	0.0142013795819731	1.02683385773398	1.2679299315377
DAGLA	1.13287724613597	0.0224989317492794	1.01775437017283	1.2610221998798
DAGLB	1.14953172520115	0.0127656791055099	1.03011910526487	1.28278679668227
DGKZ	1.12878107096397	0.0314570808454409	1.01082926670298	1.26049645388924
LPIN3	1.14389412342851	0.0173963346763416	1.0239254192036	1.27791901741439
LYPLA2	1.1429118159649	0.0179679255895429	1.02320200792527	1.27662710682207
MBOAT1	0.875274146047553	0.0210499602847105	0.781615513970781	0.980155609818042
MOGAT1	1.15624993585843	0.0273665418933159	1.01633882210226	1.31542147667573
PLA2G12A	0.846050463960799	0.00365357634517594	0.755855549693822	0.947008178822312
PLA2G2D	0.859274260268495	0.0138229774650693	0.761537768147809	0.969554348112988
PLA2G2F	1.12553233417038	0.0392189608030876	1.00586247659215	1.25943960008827
PLA2G4A	0.886308497469664	0.0375735053235789	0.791010531098217	0.993087603519397
PLCD4	1.14833137968952	0.013641857078217	1.02881410972759	1.28173296333269
PLCG1	1.16385503622236	0.00705880700090229	1.0422131096913	1.29969440294352
HMGCS2	0.874168088265509	0.0315264867421355	0.773323571093585	0.988163137793839
MVK	1.1349673823333	0.0150178746761595	1.02487518629545	1.25688569319031
ABHD5	0.861806499538127	0.0089173248119401	0.770908253868705	0.963422610821665
ADH1B	1.18622550629416	0.00213462768983927	1.06372626673465	1.3228318184737
ALDH1L1	1.13907289002252	0.0229457808945654	1.01816200211005	1.27434243872323

ARSG	0.873340664508592	0.0167530308805282	0.781613905490479	0.975832071213836
ARSI	1.21119285609693	0.00122320387943686	1.07838236150457	1.36035991224252
CA1	1.13337016909114	0.0268313102346403	1.0144668462736	1.2662098765515
CBR4	0.852157499700871	0.00508817467917959	0.761919242078706	0.953083167075897
GGT7	1.13728188145616	0.0202559893556689	1.02023922987368	1.26775175862292
GSTZ1	0.820943733916257	0.00176584500641964	0.725448443632842	0.929009663155965
NMNAT2	0.885861130094002	0.0367058003334034	0.790647647403411	0.992540665097334
ENPP1	1.15557111015719	0.0125790979133412	1.03151508636401	1.29454683531278
NME6	0.885454672124665	0.0366189513057186	0.789986334625688	0.992460175604034
NUDT6	0.863372003137217	0.0134627508956209	0.768416941249453	0.97006088203772
NUDT7	0.874845231049444	0.0187629771502573	0.782536415229991	0.978042891543921
ENOSF1	0.861842598222377	0.0139148542641436	0.76554343199435	0.970255419964441
GLYATL1	0.862973063711761	0.0223506110261372	0.760472041400387	0.97928979390311
TAP1	0.875878974400574	0.0128493813360659	0.789045328829102	0.972268575413061
ALDH4A1	1.14500734867403	0.0115232074115846	1.0308264428547	1.27183565924719
MUT	0.854260577375804	0.00797785674286205	0.760416091919832	0.95968660028749
AK3	0.888084970451266	0.0337586486606709	0.795914099603462	0.990929693461099
AK7	0.872207852735199	0.015607827283569	0.780705765627853	0.974434379591325
GMPR	0.819874797847402	0.000664458291525065	0.731278027526625	0.919205362177856
CYB5B	0.884222370534254	0.0357337798423401	0.788288419187792	0.991831392574293
CYB5D2	0.871882870304561	0.0135010075492511	0.782017446727022	0.972075166240983
CYB5R1	0.871883702361311	0.0174707563268537	0.77867489903126	0.976249769177096
CYB5R2	0.871211776566637	0.0229078761936881	0.773638806655581	

0.981090856739173
CYBRD1 1.18240879193038 0.00321651102596615 1.05769317524524
1.3218299824144
PRDX6 0.866613494245738 0.015227818543664 0.771993427171151
0.972830754739453
ADCY1 1.12290790617404 0.0229563705706323 1.01614457496726
1.24088854756598
PDE1A 1.11433851818958 0.0395146768173617 1.00521010215614
1.23531421984065
PDE1C 1.14994394305353 0.0021533602476743 1.05176022765937
1.25729328547473
PDE2A 1.10786599526551 0.0340815130105264 1.00772244260995
1.21796142625029
SLC10A2 1.09608573532424 0.0382501226916801 1.00498004606987
1.19545054041575
SLC10A7 0.8596663699656 0.00917203751565803 0.767240697454432
0.963226103753085
SLC17A7 1.13672522441411 0.0157502423866428 1.02442493717691
1.26133617889093
SLC22A2 1.20867885563891 0.000351780210990203 1.08935651091644
1.34107113826269
SLC25A17 0.841362930765262 0.00759939481035344 0.741144653894014
0.955132817253165
SLC25A27 0.881864284399985 0.03749537340907 0.783361974809749
0.992752573022413
SLC27A4 1.12129587088007 0.0371707184443765 1.00683118270021
1.24877382788319
SLC27A6 0.870486241485884 0.0163833681498397 0.777273270765122
0.974877594684711
SLC2A11 0.829310525422615 0.00196746306880029 0.736622616002305
0.933661189102809
SLC33A1 0.883289713843735 0.0340731598108533 0.787510313545272
0.990718096211061
SLC35C2 1.17860055044181 0.00260732280599955 1.05902314325671
1.31167979316295
SLC35D3 1.12159743579376 0.0382916426283618 1.00620939319729
1.25021771460691
SLC38A4 1.16486626811706 0.00114514747272053 1.06251310072014
1.27707923947225
SLC46A2 1.13694191503822 0.0196213586967598 1.02075741386276
1.26635075152594
SLC4A1 1.12196656294527 0.0190383688249141 1.01906640588019
1.23525705597169
SLC4A10 1.13588451568347 0.0224786285706105 1.0181518218004

1.26723108022136
SLC4A4 0.872613533423601 0.0192289588995387 0.778533675066768 0.97806222530929
SLC4A8 0.832400382145466 0.00167240042572466 0.742422894964506 0.933282635672279
SLC6A2 1.12978975716713 0.0168828154571231 1.02216802742435 1.24874273226495
SLC7A1 1.1513254481543 0.0163492841448385 1.02622331234332 1.2916782065113
SLC7A11 0.814630563656138 0.000756825850024465 0.723014957868121 0.917855084491575
SLC9A6 0.868809172171729 0.00856449073601774 0.78233167462537 0.964845732484481
NPC1 1.1681170222671 0.00582802244960646 1.04596047934386 1.30454008985704
SV2B 1.09894365711754 0.0373749061733984 1.00553036170843 1.20103500352489
CH25H 1.14897052514578 0.0124733535450848 1.03038251915037 1.28120697228281
CYP39A1 0.888355816144334 0.0398053707960785 0.793541709190969 0.994498520918385
GGPS1 0.838115813164685 0.00265353297556741 0.746938142698772 0.940423411420271
GALK2 1.1230914564252 0.034158411162593 1.00870945473792 1.25044373637103
PYGB 1.19423635548403 0.00112431671151838 1.07326163732186 1.32884696812477
DSE 1.14710620203484 0.0174259002579304 1.02439998241044 1.28451060263645
SRXN1 1.1640810907392 0.0149614503310522 1.02999610035432 1.31562127793533
SULT1A2 0.85388917291977 0.0175589851846107 0.749524283282291 0.972785986915117
IDO1 0.878932603438582 0.0232490663745278 0.786232722036391 0.982562159695469
FAH 0.87508684608561 0.0234644606745373 0.779693483231312 0.982151325695866
HPDL 0.862898320172308 0.0112255906935394 0.769941509221832 0.967078020911927
ARG1 1.11916898120554 0.0330127131461201 1.00912275627406 1.24121589836834
RDH5 0.876508330916021 0.0264890745287335 0.780178017393135 0.984732762315266
